# On the influence of various physicochemical properties of the CNTs based implantable devices on the fibroblasts’ reaction in vitro

**DOI:** 10.1007/s10856-015-5597-x

**Published:** 2015-10-13

**Authors:** Aleksandra Benko, Aneta Frączek-Szczypta, Elżbieta Menaszek, Jan Wyrwa, Marek Nocuń, Marta Błażewicz

**Affiliations:** AGH University of Science and Technology, Faculty of Materials Science and Ceramics, 30 Mickiewicza Ave., Kraków, 30-059 Poland; Department of Cytobiology, Collegium Medicum, Jagiellonian University, 9 Medyczna St., 30-068 Kraków, Poland

## Abstract

Coating the material with a layer of carbon nanotubes (CNTs) has been a subject of particular interest for the development of new biomaterials. Such coatings, made of properly selected CNTs, may constitute an implantable electronic device that facilitates tissue regeneration both by specific surface properties and an ability to electrically stimulate the cells. The goal of the presented study was to produce, evaluate physicochemical properties and test the applicability of highly conductible material designed as an implantable electronic device. Two types of CNTs with varying level of oxidation were chosen. The process of coating involved suspension of the material of choice in the diluent followed by the electrophoretic deposition to fabricate layers on the surface of a highly biocompatible metal—titanium. Presented study includes an assessment of the physicochemical properties of the material’s surface along with an electrochemical evaluation and in vitro biocompatibility, cytotoxicity and apoptosis studies in contact with the murine fibroblasts (L929) in attempt to answer the question how the chemical composition and CNTs distribution in the layer alters the electrical properties of the sample and whether any of these properties have influenced the overall biocompatibility and stimulated adhesion of fibroblasts. The results indicate that higher level of oxidation of CNTs yielded materials more conductive than the metal they are deposited on. In vitro study revealed that both materials were biocompatible and that the cells were not affected by the amount of the functional group and the morphology of the surface they adhered to.

## Introduction

Since their discovery in 1952, carbon nanotubes (CNTs) have been attracting increasing attention in being applied in various areas of materials science due to their outstanding mechanical properties, high chemical and thermal stability and, in some cases, very good conductivity via an electron transfer. This latter feature, characteristic to approximately one-third of all single walled carbon nanotubes (SWCNTs), and all of the multi walled carbon nanotubes (MWCNTs) is particularly interesting, as it gives an opportunity for further development in the field of miniaturization of the electronic devices. In this aspect, single CNTs are viewed as single electrodes that might be applied in many fields of science, including the so-called molecular electronics [1].

The advantage of nanotubes lies however not only in applying them as a single electrode but also, as an assembly, increasing the surface area, improving electrical conductivity, and introducing improved properties of electron-transfer reactions [2].

Coating the material with a layer of CNTs has been a subject of interest in the development of new, improved biomaterials. Such surface coatings, made of properly selected CNTs, may either be used in tissue engineering or in the field of implantable electronic devices that are used to electrically stimulate the cells’ growth or to record biochemical changes in the implant environment [2, 3]. In the literature, numerous studies report CNTs having tendency to induce differentiation, growth and proliferation of different types of tissues: nervous, connective, cardiac and vascular [3–8]. This can be caused either spontaneously by the CNTs’ physicochemical properties or by the electrical stimulation. Combining these two aspects gives a promise of significantly increasing the rate of tissue regeneration [9–11].

Fibroblasts are cells that play the key role in the processes of regeneration of every type of tissue. They have an ability to differentiate into myofibroblasts and are characteristic of the repair stage of the tissue healing process, wherein their role is to synthesize the compounds of the extra cellular matrix (ECM) [12, 13]. That is why, when considering a material aimed to be applied as an implant, regardless of its type, it is very important to test the material’s cytocompatibility with fibroblasts. These cells however can also be viewed as villains as they participate in the foreign body response (FBR), wherein they synthesize the fibrotic capsule which is aimed to isolate the foreign material from the body. This phenomenon is generally unwanted but is particularly negative in the field of implantable, long-term electronic devices as it results in failure as the quality of the registered or delivered signal is compromised. Some studies suggest that inhibiting the fibroblasts adhesion by altering the chemical composition of the material is a key to reduction of the level of fibrotic capsule formation [14]. Even though via this process the thickness of the fibrotic capsule would be indisputably reduced, one must also consider fibroblasts’ beneficial role in the processes of the regeneration of various types of tissues. Due to importance of the fibroblasts in the processes of regeneration/fibrotic capsule formation, we decided to evaluate whether there is any property of CNTs layers that influences their viability.

The goal of presented study was to generate and evaluate applicability of highly conductible material designed as an implantable electronic device, to provide an ability to monitor physiochemical properties of surrounding tissues while at the same time enable for an electrical stimulation of the cells to proliferate and differentiate. Two types of CNTs with varying level of oxidation were chosen for the study. The material was suspended in a diluent and used during the electrophoretic deposition process to generate layers on the surface of a highly biocompatible metal—titanium. Presented preliminary results describe an extensive evaluation of the physicochemical properties of the material’s surface (i.e., morphology via SEM, chemical composition via XPS, wettability and surface energy via goniometer), electrochemical evaluation of the samples and a combined in vitro biocompatibility, cytotoxicity and apoptosis study, in contact with murine fibroblasts (L929). The goal was to answer the question how the chemical composition and CNTs distribution in the layer alters the electrical properties of the sample and whether any of these properties have influence on the overall biocompatibility and stimulation of the fibroblasts’ proliferation.

## Materials and methods

### Materials

Two types of multi-walled CNTs were obtained from the NanoAmor Inc.: short-length, non-functionalized MWCNTs (stock #1213NMGS) and short, OH-functionalized MWCNTs (stock #1249YJF). The non-functionalized CNTs were subjected to a chemical reflux in the mixture of concentrated H_2_SO_4_ and HNO_3_ acids. Oxidizing in acids is expected to introduce polar functional groups to the CNTs walls, yielding charged and easily dispersible CNTs, as already described in our previous study [15]. Both the OH-functionalized and the refluxed CNTs were dispersed in the mixture of acetone and ethanol (in the 1:3 ratio, respectively), with a small addition of water. The CNTs were dissipated in the mixture of solvents with an aid of an ultrasonic processor (VibraCell, VCX 130 from Sonics & Materials, Inc.). The obtained colloids were successively used as a substrate in the electrophoretic deposition process.

Grade 2 titanium plate (in accordance to ASTM B265) was cut into 6 mm x 6 mm squares and thoroughly cleaned from organic residues by sonication in ethanol and acetone. The squares were then etched for 30 s in a 5 % HF acid and washed with distilled water until the pH of the washed-off liquid was neutral. Chemical etching of titanium is expected to remove oxide layer and increase surface roughness and alter the topography [16]. After etching titanium plates were left to dry in ambient temperature under atmospheric pressure. For the physicochemical, electrochemical and biological evaluation, pure, untreated titanium plate was chosen as a reference as it is commonly used as a biomaterial.

All reagents used in this study were supplied by Avantor Performance Materials Poland, Gliwice.

### Electrophoretic deposition

For the electrophoretic deposition process titanium square was used as an anode and stainless steel plate was used as a cathode as parts of the simple two-electrode set-up, powered by a DC power supply (TTi EL561R). The digital multimeter (Agilent 34405) connected to a personal computer was used for a real-time current density recording. The distance between electrodes was 0.5 cm and the process was carried out for 10 s, with an applied voltage of 30 V. The samples were left to dry at ambient temperature under atmospheric pressure. Accordingly, from the OH-functionalized colloid (CNT_OH_ea), the CNT_OH samples were prepared, and from the suspension of chemically refluxed CNTs (CNT_ox_ea), the CNT_ox samples were obtained. For the Electrochemical Impedance Spectroscopy (EIS) experiments, after depositing the layer on one side of the plate, the surface was left to dry before the deposition on the other side of the titanium plate was initiated.

### Physicochemical and electrochemical evaluation

Morphology of the surfaces was visualized under a scanning electron microscope (SEM, Nova NanoSEM 200, manufactured by FEI Europe Company) operating in low vacuum conditions, using an ultra-high resolution Helix detector.

Chemical composition of the layers was evaluated using an X-ray Photoelectron Spectroscopy (XPS, Vacuum Systems Workshop Ltd., England), with a 200 W Mg Ka X-ray excitation source. Electron energy analyser was set to FAT mode with pass energy of 22 eV. Energy of the spectra was calibrated assuming binding energy of the C1s to be 284.6 eV. Spectral analysis was performed using XPS Peak 4.1 software. Deconvolution of the peaks was done by fitting to a Gaussian–Lorentzian function. For the calculation of the % atomic concentrations, the area of the fitted peaks was divided by the sensitivity factors of the given elements (0.66 for oxygen and 0.25 for carbon). Due to large energy losses for the XPS method, maximum depth from which electrons can escape and be registered on the detector is no larger than 10 nm and therefore, it was assumed that only the most outer surface of the layers was analysed. The probing area of the X-ray beams is relatively high (approx. 9 mm^2^), significantly larger than any expected heterogeneities in the sample, therefore, it was assumed that one measurement generated a sufficiently representative result.

Wettability and solid state surface energy of the samples were both evaluated by a static contact angle method (DSA 10 Kruss goniometer), using ultra high quality water (UHQ, PureLab, Vivendi Water) and diiodomethane as a mean to evaluate the polar-dispersion surface tension components. For each material, three distinct samples were prepared and each of them was tested at least 10 times. The results are expressed as mean values ± standard deviation (SD).

Electrochemical properties of the samples were evaluated via an EIS. A Solatron SI 1260 Impedance/Gain-Phase analyser, coupled with a SI 1296 dielectric interface was used. The samples were measured using a two-electrode mode. The sample was placed between two platinum electrodes to provide a sufficient electrical contact and to define the actual surface of the measured plate. Conducting paste was not used. The measurements were carried out at ambient temperature, in the atmosphere of synthetic air flow, with frequencies ranging from 0.1 to 10^3^ Hz. Usage of electrolyte was omitted in order to simplify the measuring conditions and reduce the risk of oxidation–reduction reactions between the sample and an electrolyte.

### Cell culture experiments

Prior to the experiments, the plates were sterilized in the laminar flow cabinet by immersing in 70 % alcohol and irradiating each side with UV light for 30 min.

The cell–material interaction was studied in vitro using murine L929 fibroblasts (Sigma, USA). The cells were cultured in 75 cm^2^ tissue culture flasks (Nunc, Denmark), in EMEM culture medium supplemented with 10 % FBS (ATCC, USA), in a humidified atmosphere of 95 % air/5 % CO_2_ at 37 °C. All tests were conducted on cells from passages 4 and 5.

The cell suspension was obtained by adding 5 % trypsin with EDTA (HyClone, USA). After flushing and centrifuging, the cells were resuspended at 1 × 10^4^ cells/ml, seeded on a sterile biomaterial samples and placed in the wells of a 48-well culture plates (Nunc, Denmark). The cells were cultivated with biomaterials for 3, 7 and 14 days and at the given time of incubation, cell viability/proliferation, apoptosis and cytotoxicity tests were conducted.

Cell viability was tested with colorimetric CellTiter assay (Promega, USA). To determine possible cytotoxic effect of the biomaterials ToxiLight_BioAssay Kit and ToxiLight 100 % lysis reagent set (Lonza, USA) were used. The ToxiLight BioAssay is a bioluminescent assay designed to measure toxicity in mammalian cells and cell lines in culture. The ToxiLight 100 % lysis reagent, used with the ToxiLight BioaAssay, provides a total control value, proportional to the total cell number. This value may also be used to indicate the proliferation rate.

Initiation of apoptotic death of cells cultured in contact with biomaterials was examined with luminescent Caspase-Glo^®^ 3/7 Assay (Promega, USA).

All of the tests were conducted according to the manufacturer’s instructions.

The intensity of absorbance and bioluminescence in performed tests was measured using a PolarStar Omega microplate reader (BMG Labtech, Germany).

All of the tests were repeated in four separate experiments for each sample and the results were expressed as a mean ± standard deviation (SD). Significance (*P* < 0.05) was determined using an unpaired Student’s *t* test. The n value varied from 4 (Caspase-Glo^®^ 3/7 Assay) to 8 (ToxiLight and CellTiter).

## Results and discussion

### Morphology

**Fig. 1:**
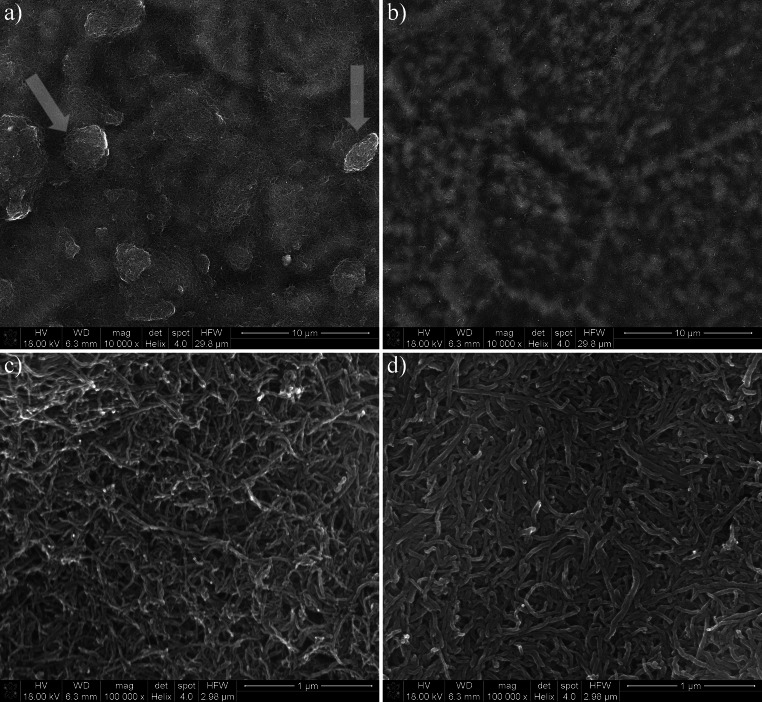
SEM observations of the CNTs layers, under two magnifications. **a** CNT_OH, mag. ×10,000; **b** CNT_ox, mag. ×10,000; **c** CNT_OH, mag. ×100,000; **b** CNT_ox, mag. ×100,000. *Arrows* indicate presence of agglomerates on the surface of the material. All images were obtained in low vacuum conditions, with accelerating voltage of 18 kV

Morphology of the layers, observed via SEM is presented in Fig. 1. The 10 k × magnification (Fig. 1a, b) reveals the overall appearance of the layer indicating the degree of homogeneity, while the 100 k × magnification (Fig. 1c, d) indicates the CNTs’ alignment within the layer. It was found that the CNT_OH layer (Fig. 1a) is composed of unevenly distributed CNTs with relatively large agglomerates clearly visible on the surface of the material (as indicated by arrows) indicating that the CNTs in the CNT_OH_ea suspension were poorly dispersed and particles in the colloid were in a flocculated state. The possible cause of such phenomenon is an insufficient amount of functional groups on the outer sidewalls of the CNTs decreasing the level of solution-particle polar-type interactions in favour of particle–particle attraction via Van der Walls forces [17]. The CNT_ox layer (Fig. 1b) was formed with evenly distributed CNTs with no larger agglomerates and no irregular topographies visible. This indicated that in the CNT_ox_ea suspension, the CNTs were well dispersed and the deposition took place in a stable state, where the particle zone close to the deposit layer mutually repels.

In both samples, titanium substrate was fully covered with a CNTs layer, indicating that the deposition was conducted long enough to cover the entire surface of the metal. However, in the CNT_ox, grain boundaries between titanium crystallites were clearly visible (Fig. 1b). Due to higher energy states of the boundaries, these regions are possibly places were the deposition of CNTs is more favoured and thus, at this places, thicker layer of CNTs might be expected, resulting in visible projection of the titanium structure. During longer times of deposition, differences in energies are equalled and there are no places of more favoured deposition, resulting in a more uniform layer [15].

Higher magnifications reveal that the surface of the CNT_OH (Fig. 1c) is composed of randomly oriented CNTs, that are not visibly adhered to one another, while in the CNT_ox (Fig. 1d), strong preference in a vertical orientation is observed, with CNTs strictly adhering to one another along their sidewalls. This result is in a good agreement with our previous study [15], where similar morphologies were observed for the layers composed of chemically refluxed CNTs, deposited from the aqueous solution. Such dense packing of CNTs may either be a result of Van der Waals attraction forces or the functional groups interactions between consecutive CNTs. As reported by Pennisi et al., an increased nano roughness is expected to decrease the fibroblasts’ adhesion and rate of their proliferation, increasing with geometrical parameters of the surface [18]. Thus, both CNTs covered samples are expected to retard fibroblasts’ growth, with a more pronounced retardation for the sample with higher surface area, namely the CNT_OH.

### Chemical composition

XPS spectrum of typical oxygen-functionalized CNTs is composed of two distinctive photoelectronic lines. One of them, with binding energy (B.E.) of approx. 284.6 eV, arises from the electrons excited from a 1s subshell of the carbon atoms (C1s), while the other, with B.E. of 532.9 eV, originates from the electrons exiting a 1s subshell of the oxygen atoms (O1s). Since some of the atoms in the sample are in different chemical states, due to the presence of the structural defects and functional groups, some shifts in the binding energies are observed, resulting in an altered shape of the spectral peaks. By deconvoluting the bands, components with different B.E. can be determined and, as a consequence, most abundant chemical states can be identified.

**Fig. 2:**
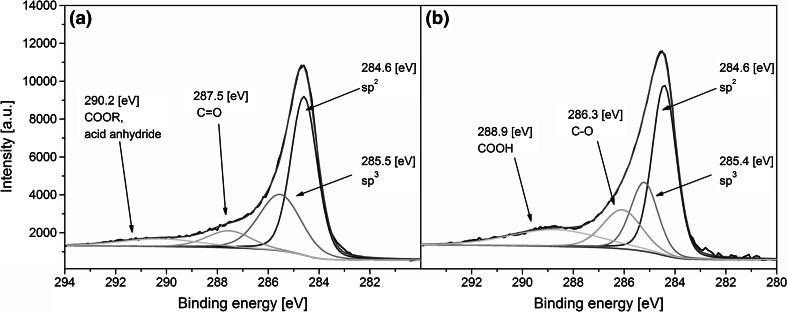
Deconvoluted, high resolution C1s XPS spectra of the CNT_OH (**a**) and CNT_ox (**b**)

Chemical states of the carbon atoms within the CNT_OH and CNT_ox samples are identified in Fig. 2a and b, respectively. In both spectra, the C1s band is composed mainly of electrons with B.E. of 284.6 and 285.5 eV, excited from the carbon atoms in the sp^2^ and sp^3^ hybridization, respectively [19]. Carbon atoms in the sp^2^ hybridization are the ones that are in the honeycomb lattice, while the sp^3^ hybridization arises from the presence of various defects in the graphene structure, including OH groups [20]. In the spectrum of the CNT_ox sample, the third prominent peak is the one with B.E. of 286.3 eV, attributed mainly to the presence of the C–O bonds [21, 22], while in the CNT_OH this band is shifted towards higher B.E., indicating formation of double bonds between the carbon and oxygen atoms [20, 23]. In the case of the CNT_ox, the last distinctive peak, observed at 288.9 eV is attributed to the presence of carboxyl functional groups. Meanwhile, for the CNT_OH, this band is slightly shifted towards higher binding energies. As some studies suggest, such shift might indicate formation of carbonates and/or acid anhydride between the functional groups of consecutive nanotubes [23].

**Fig. 3:**
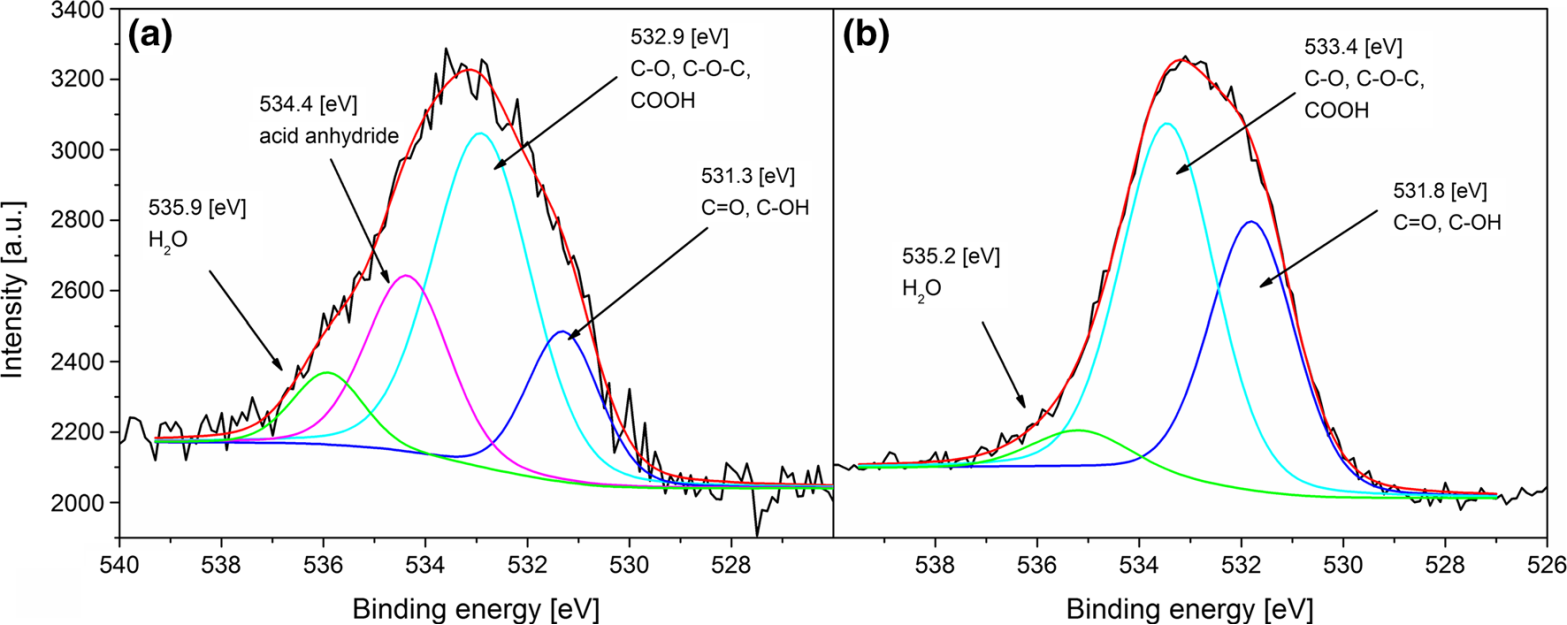
Deconvoluted, high resolution O1s XPS spectra of the CNT_OH (**a**) and CNT_ox (**b**)

Since many of carbon containing functional groups have very similar binding energies, identification of chemical species only by the deconvolution of the C1s band may be ambiguous. Thus, in order to provide more definite results, an additional deconvolution of the O1 s band was performed and is given in Fig. 3. In both cases, binding energies of electrons exited from the oxygen atoms are chemically shifted, resulting in an irregular line shape of the band. In the CNT_OH (Fig. 3a), the peak with smallest B.E. was observed at 531.4 eV. In the literature, this band is usually connected to the presence of double bond between oxygen and carbon atoms [21–23]. However, in the case of the CNT_ox (Fig. 3b), this band revealed a slight shift towards higher binding energies (531.8 eV), which can be related to the presence of hydroxyl functional groups [24, 25]. Another compound of the O1 s band in the CNT_OH spectrum had a B.E. of 532.9 eV, while in the spectrum of the CNT_ox, it was shifted to 533.4 eV. While lower B.E. are most often ascribed to the presence of a single C-O bond [25, 26], higher energies can also be attributed to the presence of various functional groups containing a C=O bond [24]. The shape of the O1s band in the CNT_OH spectrum revealed the presence of oxygen functionality with B.E. of approx. 534.4 eV, however spectrum of the CNT_ox had no corresponding signal. This band may have originated from either acid anhydride [22] or carbonates [21] indicating good agreement with the C1s deconvolution, where no chemical groups with B.E. of 290.2 eV was observed in the case of the CNT_ox sample. In both spectra, a small peak at approx. 535 eV, attributed most likely to a physisorbed water, was observed [19].

A quantitative evaluation of the chemical functionalities present in both of the tested samples is presented in Table 1. When compared to different studies, both samples contain relatively high share of oxygen [22, 23, 27]. For example, Chiang et al. reported a 7.8 % percent of oxygen atoms after refluxing the CNTs for 2 days in conditions similar to ours—a boiling mixture of sulphuric and nitric acids [23]. In materials obtained in our study the overall amount of oxygen atoms is 7.6 % for the as-received CNT_OH and more than twice fold larger (15.5 %) for the CNT_ox. Increased oxygen fraction in our material indicates the chemical treatment applied is far more oxidizing than the treatment proposed by the NanoAmor, which, according to the manufacturer is also a chemical reflux. The efficiency of the process applied was also much higher than reported in the literature [23]. As a result, more easily dispersible CNTs were obtained, giving a better chance for deposition in the stable state, as indicated by high homogeneity of the CNT_ox layer, when compared to the surface of the CNT_OH (Fig. 1b and a, respectively). In the CNT_OH, deconvolution of the C1s band suggested that a very large share of carbon atoms (30 %) were either in a sp^3^ hybridization (indicating high level of defects) or bonded with hydroxyl functional group. Deconvolution of the O1 s band revealed that only 17 % of all of the oxygen atoms within the CNT_OH sample were related to the presence of hydroxyl groups and therefore the increased area of the 285.8 eV band was concluded to be attributed mostly to the presence of structural defects rather than functional groups. Majority of oxygen atoms present in the CNT_OH sample was either in carbonyl or acid anhydride functionality. This indicates that agglomerates visible on the surface via SEM (Fig. 1a) may have formed not only by the Van der Waals interactions between the tubes but also by strong chemical interactions between the functional groups of neighbouring CNTs. On the other hand, in the CNT_ox material, a smaller amount of carbon in the sp^3^ hybridization was observed, in favour of large share of the C-O bonds and carboxyl functional groups. No bands connected with the presence of acid anhydride were observed suggesting the good dispersion and a consecutive high homogeneity of the deposited layer (Fig. 1b) in the absence of those functionalities. These results imply the applied procedure of chemical reflux in the mixture of concentrated acids preferably oxidize defects in the structure of CNTs most likely due to the differences in the energy state. In these places, carbon of higher oxidation states is readily formed, yielding easily dispersible CNTs.

**Table 1:** Chemical composition of the CNT_OH and CNT_ox samples, evaluated through band fitting of the high resolution XPS spectra

Binding energy (eV)	Chemical species	CNT_OH (wt%)	CNT_ox (wt%)
*C1s total content*		92.4	84.5
284.6	C=C (sp^2^)	52.9	45.7
285.8	C–C (sp^3^), C–OH, C–O–C	30.0	20.8
286.3	C–O	0.0	16.8
287.5	C=O	9.5	0.0
288.9	COOH	0.0	16.7
290.2	COOR, C(O)–O–(O)C	7.7	0.0
*O1s total content*		7.6	15.5
531.3–531.8	C=O, C–OH	17.3	38.5
532.9–533.4	C–O, C–O–C, COOH	51.4	55.0
534.4	C(O)–O–(O)C	23.2	0.0
535.2–535.9	H_2_O	8.1	6.4

A concentration of carboxyl groups found in CNT_ox sample during current study is higher (16.7 %) than reported in our previous study (9.7 %) [15]. Since the starting material was the same, this indicates that the applied mixture of solvents may favour deposition of CNTs with higher amount of COOH groups. In our previous study we discovered that higher concentrations of carboxyl groups may reduce the osteoblasts’ adhesion. Contrary to that, Faucheux et al. [28] found that fibroblast prefer the COOH terminated self-assembled surfaces over the OH terminated ones. At the same time, Kamath et al. found that an increased amount of COOH groups on the surface of a biocompatible material may reduce the formation of fibrotic capsule in vivo [29]. The overall amount of carboxyl and hydroxyl groups in the CNT_ox is significantly higher than in the CNT_OH and, based on the literature, differences in fibroblasts’ viability and proliferation are expected to be observed for these two samples.

### Wettability and surface energy

**Fig. 4:**
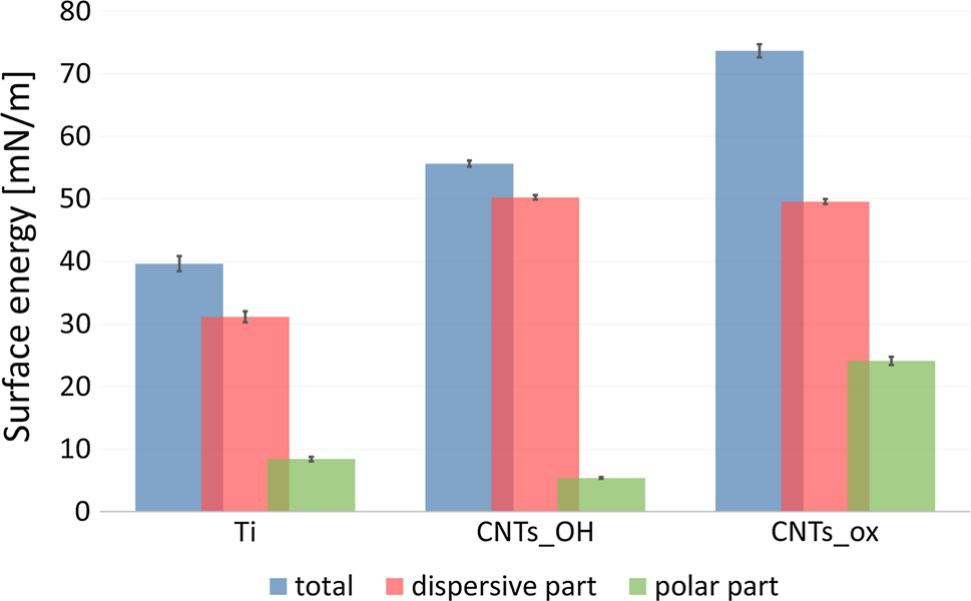
Total surface energy and its dispersive (σ^D^) and polar (σ^P^) components for all of the tested samples

Pure, untreated titanium has an average water contact angle of 76.2° ± 3.7° and for the CNT_OH sample, the contact angle of 68.6° ± 2.3° was measured. The CNT_ox however, has exhibited an increased wettability, manifested by a drop in a contact angle value to 29.9° ± 5.5°. This value is very comparable to the one we have obtained in our previous study, where the same CNTs were deposited from water, for 30 s [15] and comparable to the material obtained by Chiang et al. [23]. The described results suggest that upon the deposition, bulk level of polar component in the highly oxidized CNTs remains the same, regardless of time of deposition and the solvent applied. In Fig. 4, calculated values of surface energy, together with its dispersive (σ^D^) and polar (σ^P^) components are presented. It was found that both types of CNTs increase the total surface energy of titanium due to the presence of different functional groups and increased nano roughness. In both types of CNTs surfaces, a comparable increase in the σ^D^ was observed, attributed to the presence of large share of non-polar C–C chemical bonds. At the same time, significant differences concerning the σ^P^ were found. In the case of the CNT_OH, a slight decrease in the polar part, when compared to titanium was observed. In titanium, this part was most probably attributed to a spontaneously formed layer of titanium oxide. Meanwhile, in the case of CNT_OH, small amount of polar groups, as already proven by the XPS study (Table 1) and increased nanoroughness yield a slight decrease in the σ^D^. At the same time, in case of the CNT_ox, the polar component was very large, arising from high level of oxidation (15.5 %), as proved by the XPS study (Table 1).

### Electrochemical evaluation

**Fig. 5:**
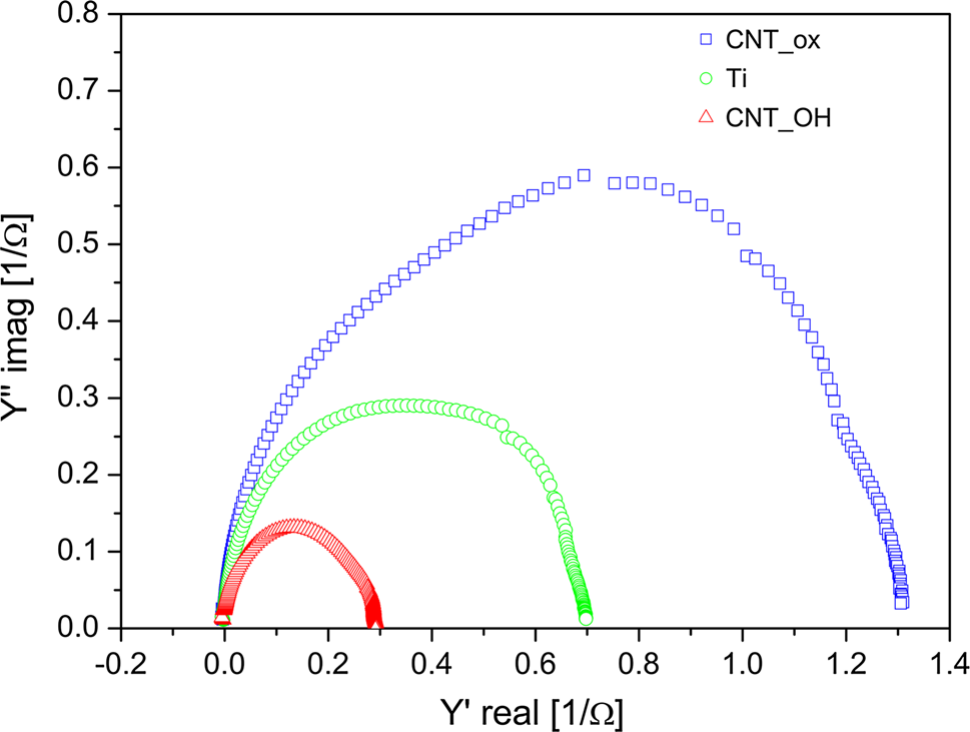
Nyquist plot of the CNT_ox, CNT_OH and titanium obtained during the EIS measurements

**Fig. 6:**
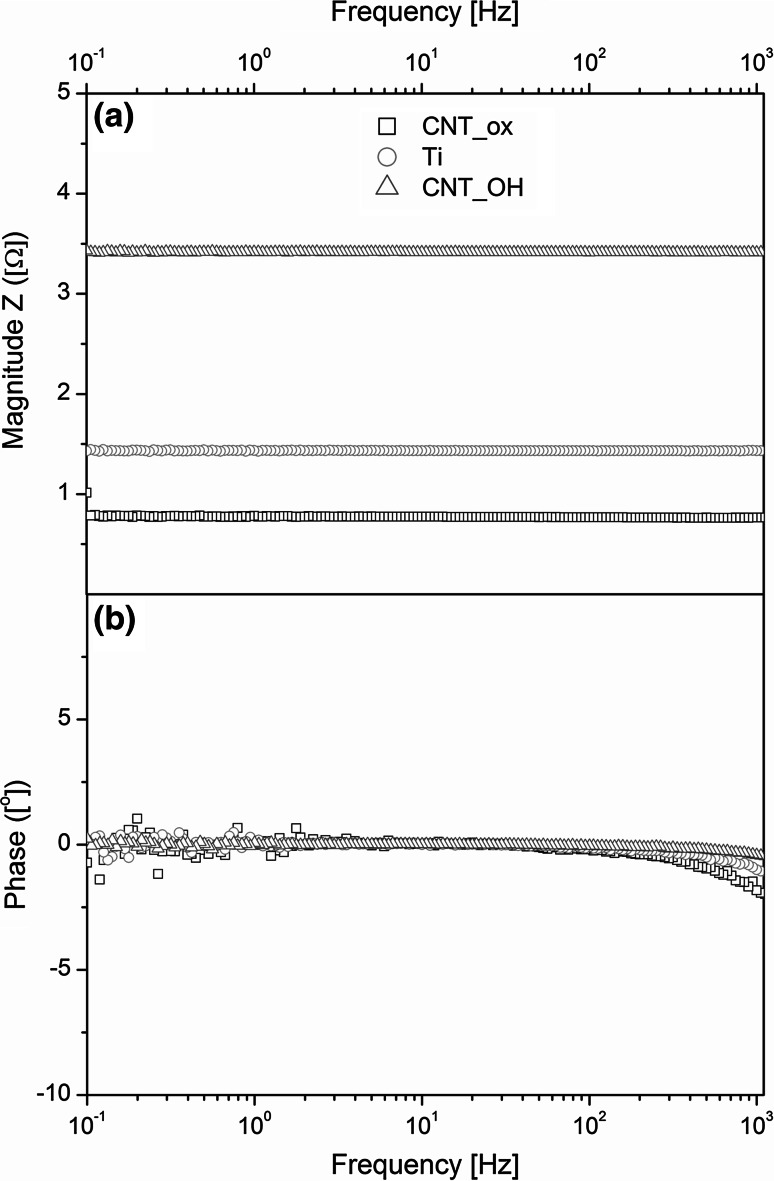
Bode plots of the CNT_ox, CNT_OH and titanium obtained during the EIS measurements. Absolute impedance (**a**) and phase shift (**b**) versus log of angular frequency

The EIS experimental data of the tested materials are presented in Fig. 5 and Fig. 6, in the form of the Nyquist and Bode plots, respectively. The Nyquist plots represent the conductance (Y′) as a function of susceptance (Y″), while the Bode plots are used to express the value of impedance magnitude and phase angle as a function of frequency in a logarithmic scale. The results indicate that the dominant conductivity mechanism is the electrons-based transport. This implies that the titanium oxide underlayer, expected to form spontaneously during an EPD process, is either not formed or its thickness is negligible (since titanium oxide is a semiconductor, it should significantly change the character of the Nyquist plot and reduce the conductance). The higher the value of the admittance, the more conductive is the sample. Remarkably, in the case of the CNT_ox, the sample seems to be even more conductive than the metal itself which is a direct result of a decreased resistance of this material as observed in the resistance Bode plots (Fig. 6a).

In Bode plots the actual values of absolute impedance (Fig. 6a) and phase angle shift (Fig. 6b) are plotted against an increasing log of angular frequency of the electrical field. Constant impedance [Z (Ω)] and phase angle shift (°) with increasing input frequency indicate that the capacity of the circuit is negligible. The presence of a significant parallel capacitor would be manifested by a decreasing value of the impedance, connected with a negative angle shift, as a function of increasing frequency. At the same time, no electromagnetic inductance occurs, as this would be observed as a positive angle shift with an increasing frequency of the input current. Therefore, the CNTs are presumed not to introduce any additional electronic elements to the circuit, other than a possible additional resistor.

As observed in the Nyquist (Fig. 5) and Bode plots (Fig. 6a) the conductivity of the CNT_ox sample is much higher than the one of the CNT_OH. Since the surface of the CNT_ox is formed from more oxidized CNTs, their conductivity was expected to be lower than in the case of less oxidized CNTs in the CNT_OH layer as oxygen-containing groups are generally observed to slow down the rate of heterogeneous electron transfer [30]. The representative tube from the less oxidized layer is expected to be more conductive. In our case however, we have measured the electrical properties of the whole sample, composed of titanium covered with a layer of CNTs. Our results indicate that not only the structure of a single tube is important but also the way it interacts with other tubes and with the surface of titanium. As observed by the SEM investigation (Fig. 1), the CNT_ox sample is composed of densely packed tubes that are well adhered to one another along their sidewalls and also, most probably, well adhered to the surface of titanium, likely facilitating the electron transfer. On the contrary, the surface of CNT_OH sample is formed of loosely and randomly distributed tubes, creating randomly distributed agglomerates, between which porous sites are also visible (Fig. 1a). Such uneven packing may have resulted in an increased resistivity, due to the reduced number of pathways for the electron transfer. A significant decrease in conductivity may also indicate poor adhesion to the surface of titanium, although in order to prove that, further studies are required.

More interestingly, the CNT_ox material revealed an increased conductivity when compared to pure titanium. In our previous study, we reported preparation of a material composed of oxidized CNTs, deposited on the surface of titanium, with a conductivity comparable to pure titanium [15]. In surface science, such a phenomenon is usually regarded as a success, since any additional coating is expected to decrease the conductivity of metal by adding an additional resistance to the circuit. This might suggest that the CNTs are not only well adhered to the surface of titanium and to each other (as observed in Fig. 1d), but may also decrease the level of electron scattering at the grain boundaries, facilitating the electron transfer between both components (CNTs and titanium). This is illustrated in Fig. 1b, where the CNT are well adhered along the grain boundaries of the metal, facilitating charge-transfer process at the electrode contacts. Another possible explanation of such a phenomenon is that the oxidized tubes, forming the dense surface of the CNT_ox sample, isolate titanium substrate from the influence of oxidizing factors. As a result, thinner oxide layer is formed in this material than in the uncoated titanium. Since oxide layer introduces an additional resistivity to the equivalent circuit analogue, in the case of the CNT_ox sample conductivity is higher than in pure titanium. However, in order to confirm any of these hypotheses, further studies need to be carried out.

The given results imply that the CNT_ox material is a promising candidate for an implantable electronic device, due to its conductivity. High conductivity of the electrode (and therefore its low impedance) is an essential parameter to efficiently record the signal or stimulate the tissue by reducing the threshold level [31].

### Cell culture experiments

**Fig. 7:**
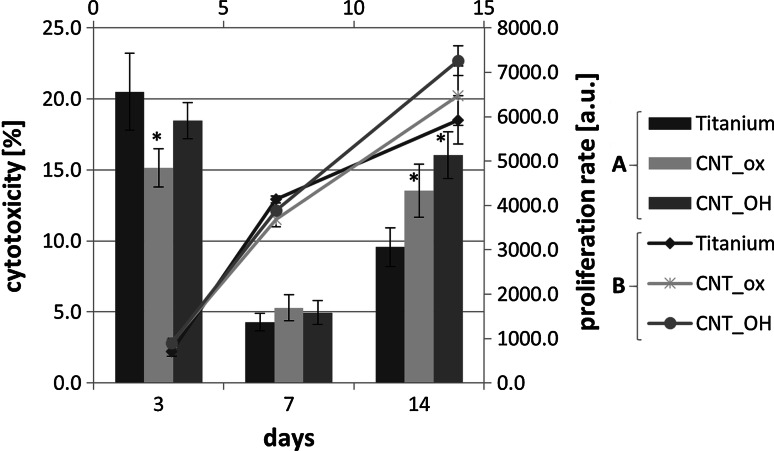
Proliferation rate (*A*), and cytotoxicity (*B*) in cell culture (L929) exposed to CNT_OH, CNT_ox and Ti samples for 3, 7 and 14 days. The data are expressed as mean ± SD from 5 to 8 measurements. *Significant difference compared to controls (cells cultured without CNTs = Ti plate) according to Student’s *t* test (*P* < 0.05)

By using the ToxiLight 100 % lysis reagent, total number of cells growing on the material can be obtained and expressed as a proliferation rate, as presented in Fig. 7. It was observed that between the 3rd and 7th day of the experiment, a continuous and almost constant growth of cells’ number is observed in all of the tested materials. In the 7th day, a total number of cells is comparable between all of the tested samples, with a slight prevalence of titanium, when compared to both of the CNTs covered surfaces. After a prolonged culture however, at 14th day, a significant change in this tendency was observed: at that time, the total number of cells on both the CNT_OH and CNT_ox samples was higher than on pure Ti. As a consequence, a different curve of the proliferation rate was observed: in titanium, after the 7th day, the multiplication of cells has slowed down. Meanwhile, in both of the CNTs covered samples, the growth rate remained almost constant during the whole length of the experiment. The given results suggest, when compared to titanium, the surface of CNTs reduces the proliferation rate of fibroblasts in the first period of culture and increases it after the 7th day. This may suggest that the surface of CNTs is slightly less favourable for the initial adhesion than the surface of titanium. Since fibroblasts are adherent cells, if their adhesion is delayed, the activation and stimulation of the processes responsible for proliferation is prolonged. And, as reported by Pennisi et al., an increased nanoroughness is expected to reduce the fibroblasts’ adhesion [18]. Interestingly however, upon being adhered, fibroblasts are found to be stimulated by the CNTs to more extent than the surface of titanium. This result is with good agreement with the literature, where CNTs were found to be highly favourable for the growth and proliferation of various types of cells [3–8].

For the CNT_OH and CNT_ox samples, the physicochemical properties such as wettability and surface energy were different than for the Ti sample (Fig. 4), i.e., the surface energies were higher for the samples covered with CNTs (γ = 55.6 mN/m and γ = 73.7 mN/m, for CNT_OH and CNT_ox, respectively) than pure Ti (γ = 39.6 mN/m). Also, the wettability of the samples was found to be lower than the wettability of pure titanium. Most likely, the main factor altering the cells behaviour cultured on the CNTs surfaces when compared to titanium was higher surface energy and increased nanoroughness of the former. Increased nanoroughness resulted in an increased number of potential areas prone to interaction with the cells, while higher surface energy energetically enhanced the adhesion of various molecules including proteins.

Another interesting result observed was a significant difference in cell number between the surfaces made of different type of CNTs, especially after 14 days of culture. Since these materials have different surface morphology, chemical composition, wettability and surface energy, it is difficult to point out one specific reason for that phenomenon. We believe that at a 14th day of culture, a potential influence of the increased nanoroughness of the CNT_OH sample on the fibroblasts functions was decreased, since there was no influence of an initial adhesion reduction [18]. Thus, at that time point, differences in fibroblasts’ proliferation rate may have been governed by different chemical composition of the samples and an increased amount of COOH species in the CNT_ox [28].

At the 14th day of culture, the number of the fibroblasts increased in all of the tested materials, when compared to the 3rd and 7th day of culture. This suggests that all of the tested materials stimulate cells’ proliferation. However, the 100 % lysis test does not differentiate between the live, the apoptotic and the necrotic cells, and thus, no information about the condition of the cells and the overall cytotoxicity of the materials is provided. For this purpose the ToxiLight assay (ToxiLight™ BioAssay Kit, Lonza) was carried out. The ToxiLight™ BioAssay Kit is a bioluminescent, non-destructive cytotoxic assay designed to measure the release of the enzyme, adenylate kinase (AK), from the damaged cells. AK is a robust protein present in all eukaryotic cells, which is released into the culture medium when the cells die. The enzyme actively phosphorylates ADP to form ATP and the resultant ATP is then measured using the bioluminescent firefly luciferase reaction. The results obtained using the ToxiLight™ BioAssay Kit are expressed as a percentage of the total number of cells, obtained via the ToxiLight 100 % lysis test (Fig. 7).

High level of cytotoxicity after the 3rd day of culture for all the samples was likely due to the fibroblast culture set up, when cells can be damaged during handling and the non-adhered cells spontaneously die. Up to 3rd day, there was no changing or refilling of culture medium and therefore, these cells were still present in the culture well and likely contributed to an increased level of biomaterials’ cytotoxicity. Interestingly, cytotoxicity of the CNT_ox is found to be slightly but significantly lower than cytotoxicity of both the CNT_OH and titanium. In the CNT_OH, geometrical parameters of the surface were expected to be higher than in the CNT_ox as they contain a lower amount of carboxyl groups—these two factors may have contributed to the decreased level of initial adhesion and a consecutive death of non-adhered cells [18, 28]. On the other hand, higher level of titanium cytotoxicity indicates that this surface was less favourable for initial adhesion than the surface with CNTs. On day 7, the cytotoxicity of all of the tested samples decreased significantly, which may prove that in that first period of time, cell death was caused by their damage during setting up the culture, rather than being a result of any negative effects of the samples (Fig. 7). On the 7th day of culture the cytotoxicity for all samples did not exceed 3 %, proving an extremely high biocompatibility of all of the tested materials. No significant differences in the cytotoxicity of all of the tested samples were observed, indicating that the surface properties affect only the initial adhesion of fibroblasts. After the culture medium was replaced and the non-adherent dead cells were removed, samples did not affect the viability of the adhered cells. However, at the 14th day of the culture, the cytotoxicity of all of the tested materials has increased rapidly. Highest level of cytotoxicity is observed in the CNT_OH sample, slightly lower in the CNT_ox and the lowest in the Ti sample. Since an identical relationship is observed for the cells’ growth rate, these results are most probably a direct consequence of an increased number of cells on the samples covered with CNTs, as observed from the proliferation rate. The high growth rate of fibroblasts may contribute to contact inhibition [32] of cell growth and their dying, and therefore a higher level of cytotoxicity of the CNTs covered materials did not necessarily indicate an adverse effect of the nanotubes and their surface chemistry. Despite exhibiting a higher cytotoxicity level than titanium, its values were still below 17 %, ranking the materials, according to the ISO standards as non-cytotoxic [33].

**Fig. 8:**
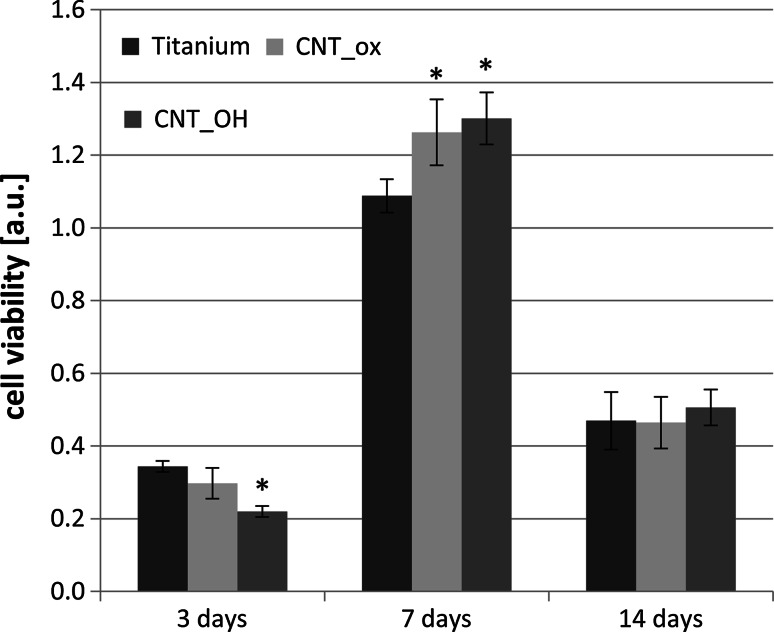
Cell viability in contact with CNT_OH, CNT_ox and Ti after 3, 7 and 14 days of culture. The data are expressed as mean ± SD from 8 to 11 measurements. *Significant difference compared to controls (cells cultured without CNTs = Ti plate) according to Student’s *t* test (*P* < 0.05)

The results obtained using the viability test (CellTiter assay) (Fig. 8) confirmed the observations from cytotoxicity assay. For all of the tested samples, the highest level of cell viability was observed after the 7th day of culture and similarly the level of cytotoxicity in this period of time was lower, confirming the assumptions made from the cytotoxicity results on the 3rd day (an amount of live cells is decreased due to the presence of the damaged cells as a leftover from the culture start up and a slightly lower viability of cells growing on the CNT_OH is attributed to the surface properties of the sample, reducing the fibroblasts’ adhesion). On the 7th day, the viability of cells had increased as the dead cells were washed off during the change of the medium and live cells were in good condition and adhered to the surface of the material. On the 14th day the viability of cells was reduced due to their high density inducing their apoptosis. Analysis of cell viability at the 14th day of culture for two types of samples modified with CNTs didn’t reveal significant differences between them. Moreover, the cell viability on analysed samples was at the similar level as for the control sample and the differences were not significant. Thus, it was concluded that the CNTs layers are biocompatible and that, after the initial adhesion, surface properties of the sample do not affect fibroblasts’ viability and proliferation.

**Fig. 9:**
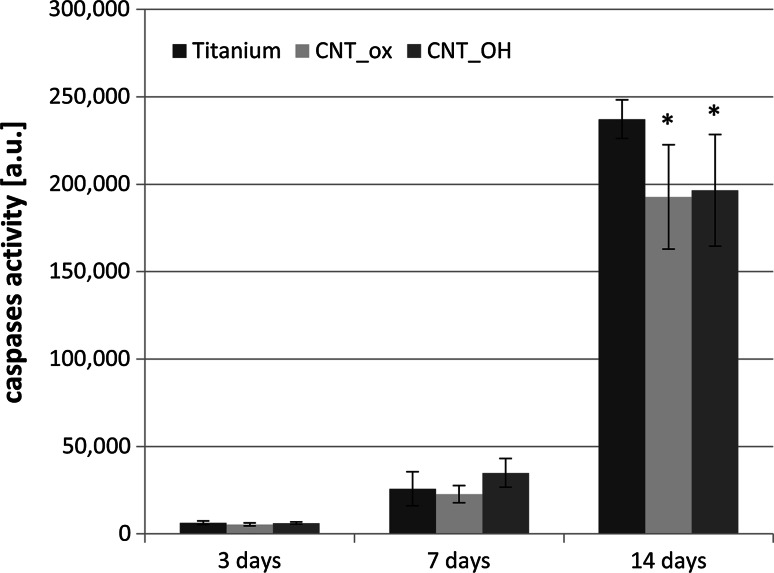
Caspases activity in contact with CNT_OH, CNT_ox and Ti after 3, 7 and 14 days of culture. The data are expressed as mean ± SD from 8 to 11 measurements. *Significant difference compared to controls (cells cultured without CNTs = Ti plate) according to Student’s *t* test (*P* < 0.05)

The value of cytotoxicity, determined using ToxiLight test, provided information on the number of dead cells, without distinguishing between apoptosis or necrosis. In order to determine to what extent cell death was caused by the apoptosis, the caspase 3/7 activity was measured (Fig. 9). The apoptosis is a naturally programmed death of the cells. In the case of healthy, adherent cells, their apoptosis is induced mostly by contact inhibition and reduction of the overall contact area with the surface. In our experiment the highest caspase activity was observed after 14 days of culture for all of the analysed samples, especially for pure titanium. Since on titanium a lower number of cells was found on day 14, this result was somewhat interesting as it indicated the surface of titanium triggers the cells’ apoptosis to a higher extent than the CNTs and that apoptosis was not necessarily related to the contact growth inhibition. The increase in caspase activity in the subsequent periods of culture for all samples indicated that the decreased viability (Fig. 8) and increased cytotoxicity (Fig. 7) were due to natural death of cells, induced by contact inhibition of growth and detachment of cells that rapidly proliferate at the surface of the samples, rather than the adverse cytotoxic reaction of the material.

As to summarize, despite having different morphology, chemical composition and electrochemical properties, the CNT_OH and the CNT_ox samples did not reveal significant differences in affecting the growth and viability of fibroblasts. At an initial step, less carboxyl groups and increased nanoroughness of the CNT_OH were found to slightly decrease the viability of cells, likely by reducing their adhesion [18, 28]. However, after a prolonged culture, this effect was dismissed, as the growing cells synthesized their own ECM, affecting the surface properties of the sample and introducing more favourable conditions for cells’ growth. After a prolonged culture, CNTs were found to stimulate the fibroblasts’ proliferation to higher extent than titanium and this result was in good agreement with the literature, where CNTs were found to be highly favourable for the growth and proliferation of various types of cells [3–8]. An increased proliferation rate most probably led to contact inhibition of growth of fibroblasts and a successive death of cells, manifested by an increased cytotoxicity, which was still below the threshold level where sample is regarded as cytotoxic [33]. Interestingly, titanium was found to trigger the cells’ apoptosis in higher level than the CNTs, indicating an additional factor inducing the cells’ apoptosis other than the contact inhibition.

## Conclusion

In the reported study, two types of CNTs were deposited on the surface of titanium, in order to obtain a highly biocompatible biomaterial to be used as a novel implantable electronic device, used to stimulate the regeneration of various tissues. The studies proved that the level of oxidation of the CNTs has a major impact on the outcome electrochemical properties and overall surface morphology of the obtained materials. Surprisingly, it was discovered that higher level of oxidation of CNTs yielded materials that were more conductive than the titanium metal they were deposited on. This is likely due to good adhesion of the CNTs, both to one another and to the titanium substrate, facilitating the electron transfer. The less oxidized CNTs on the other hand formed a loosely distributed layer, which exhibits a decreased level of conductivity, when compared to pure titanium. In vitro study in contact with fibroblasts indicated both materials being biocompatible. The layer of CNTs with an increased nanoroughness and higher share of carboxyl groups (CNT_OH) was suspected to exhibit a reduced level of initial adhesion, manifested by lower viability of fibroblasts. After an initial set-back however, the cells did not differentiate the amount of the functional group and the morphology of the surface they are adhered to and both types of CNTs stimulated the proliferation of cells to a greater extent than titanium.

In our study, two novelties in the field of applying CNTs in biomedicine are reported. First of all, a careful, comparative evaluation of two types of CNTs films on surface of titanium were performed and paired with the biocompatibility studies. To this day, similar studies have been performed mainly on the CNTs suspension [34, 35]. Moreover, the produced material exhibited electrical properties that are superior to a metal substrate. This result indicated an interesting possibility of improving the properties of present implantable electronic devices. Further studies will need to be conducted to further prove our hypothesis but the obtained results seem very promising.

It is important to mention that the electrochemical properties of the new surfaces were evaluated in simplified conditions that don’t exactly mimic the natural organism. In future, further experiments concerning behaviour of the sample in more aggressive environment (increased temperature, usage of an electrolyte) are necessary to further evaluate the material’s biofunctionality. In addition the experiments involving culturing cells on the CNT_ox sample, under the influence of an electronic field, are planned to be performed to confirm whether the facilitation of tissue regeneration by our materials is feasible.

The future studies should also include the evaluation of the susceptibility of the material to the influence of formation of fibrotic capsule, as such capsule is the most common reason of failure of an implantable electronic device [36].
